# A Comparative Study on the Postoperative Analgesic Effects of the Intraperitoneal Instillation of Bupivacaine Versus Normal Saline Following Laparoscopic Cholecystectomy

**DOI:** 10.7759/cureus.14151

**Published:** 2021-03-27

**Authors:** Shashikanth Vijayaraghavalu, Ezhil Bharthi Sekar

**Affiliations:** 1 General Surgery, ACS Medical College Hospital, Chennai, IND; 2 Anaesthesiology, ACS Medical College Hospital, Chennai, IND

**Keywords:** bupivacaine, laparoscopy, cholecystectomy, analgesia, intraperitoneal, postoperative pain, shoulder pain, nausea, vomiting, local anesthetics

## Abstract

Background

Laparoscopic cholecystectomy is widely performed, and postoperative pain is an important factor in patient morbidity during recovery. Various modalities for postoperative pain relief have been proposed, with varying levels of success such as intravenous or intramuscular non-steroidal anti-inflammatory drugs (NSAIDs) and opioids, infiltration at the incision site with local anesthetics, intraperitoneal infiltration of local anesthetics, intraperitoneal infiltration of local anesthetics with adjuvants, regional anesthesia techniques such as epidurals and nerve blocks. The study was aimed to evaluate the efficacy of intraperitoneal instillation of bupivacaine and normal saline on postoperative analgesia, postoperative nausea, and vomiting after laparoscopic cholecystectomy.

Methods

This prospective, controlled, and randomized study included 60 American Society of Anesthesiologists (ASA) I and ASA II patients, aged 18-50 years, who were scheduled for laparoscopic cholecystectomy under general anesthesia. The patients were classified randomly into two groups with an equal number of participants: Group B received intraperitoneal instillation of 30 ml of plain bupivacaine 0.5% and Group N received 30 ml of normal saline. Postoperative pain was recorded using the visual analog scale (VAS) for 24 hours after surgery. Postoperative shoulder pain, nausea, vomiting, and the time taken to request rescue analgesia were noted.

Results

Patients receiving intraperitoneal bupivacaine showed a significant reduction in postoperative pain for the first six hours postoperatively (P = 0.04); moreover, the time taken to request rescue analgesia requirement was prolonged (P = 0.04). Side effects, such as nausea and vomiting, were similar between the two groups (P = 0.1 and p = 0.09, respectively) while shoulder pain was significantly lower in the bupivacaine group (P = 0.04).

Conclusion

Bupivacaine is effective in reducing postoperative pain, and it prolongs the requirement time for rescue analgesia. It also reduces the incidence of shoulder pain but does not decrease postoperative nausea and vomiting.

## Introduction

Laparoscopic cholecystectomy is widely performed, and it has replaced open cholecystectomy as the gold standard for cholelithiasis. Laparoscopic surgeries are favored over open surgeries because they have a number of advantages such as reduced postoperative pain and analgesic requirement, improved postoperative respiratory function, rapid return of gastrointestinal function, a reduced stress response to surgery and recovery time, less postoperative wound infection, and improved cosmetic appearance [1-3].

Postoperative pain is an important factor contributing to patient morbidity during recovery. After laparoscopic cholecystectomy, pain can arise from the incision site (somatic pain), visceral structures (visceral pain) [4], and shoulder tip (referred pain from the subdiaphragmatic region). Visceral pain occurs due to the stretching of the parietal peritoneum, the release of inflammatory mediators of pain, and the irritation produced by blood. Referred shoulder pain is often mild and is due to the irritation of the diaphragm by residual gas [5].

Various modalities are used for postoperative pain relief, including intravenous or intramuscular NSAIDs [6] and opioids [7], infiltration at the incision site with local anesthetics [8], intraperitoneal infiltration of local anesthetics [8], local anesthetics with adjuvants [9], and regional anesthesia techniques such as epidurals and nerve blocks [10-11]. These have been found to have variable rates of success.

The present study was conducted to evaluate the effects of the intraperitoneal instillation of bupivacaine and normal saline on postoperative analgesia and postoperative nausea and vomiting (PONV) in patients undergoing laparoscopic cholecystectomy.

## Materials and methods

This randomized, prospective, double-blind study was conducted in ACS Medical College Hospital from June 2020 to December 2020, after obtaining approval from the Institutional Ethics Committee, and written informed consent was obtained before surgery from all the patients participating in the study.

The study population consisted of 60 male and female patients, ranging in age from 18 to 50 years, with either an American Society of Anesthesiologists (ASA) I or ASA II physical classification status that was scheduled for elective laparoscopic cholecystectomy under general anesthesia. Exclusion criteria were patient refusal, pregnant or lactating patients, a body mass index (BMI) of > 40, allergy to local anesthetics, patients with cardiac, pulmonary, neurological, and renal diseases, any patient who needed conversion to open cholecystectomy, previous history of abdominal surgeries, and duration of surgery of > two hours.

The patients were randomized into two groups of 30 patients each using sequentially numbered envelopes. To maintain the double-blind nature of the study, the randomization and preparation of the drug for injection were done by an anesthesiologist not involved in the study. Group N received 30 ml of normal saline and Group B received 30 ml of 0.5% plain bupivacaine. The postoperative follow-up of the study participants was done by the co-author who was blind to the patient group assignment.

All the patients fasted overnight, and the preoperatively enrolled patients received instructions about the visual rating scale employed in this study to indicate their pain. On arrival to the operating room, an 18-gauge intravenous (IV) cannula was secured and IV fluids at 2 ml/kg were started. The Association of Anaesthetists of Great Britain and Ireland (AAGBI) standards for monitoring started and baseline vital parameters were noted.

General anesthesia with endotracheal intubation was administered in all patients using propofol 2 mg/kg, fentanyl 2 ug/kg, and vecuronium 0.1 mg/kg after pre-oxygenation with 100% oxygen for three minutes. The airway was secured with an appropriately sized, cuffed endotracheal tube. Anesthesia was maintained with 50% oxygen and 50% air with 1% isoflurane and intermittent doses of muscle relaxant. Minute ventilation was adjusted to maintain End-tidal CO_2_ (EtCO2) between 35 and 40 mmHg. No other intraoperative analgesic supplementation was used. CO_2_ was used to institute pneumoperitoneum, and the intra-abdominal pressure was maintained between 12 and 14 mmHg. The patients were placed in a 15-30-degree reverse Trendelenburg position with a left lateral tilt.

At the end of the surgery, the study drugs, as per group allocation, were instilled in equal amounts into the sub-diaphragmatic space and intraperitoneally under direct vision, and the patients were placed in the Trendelenburg position. The pneumoperitoneum was removed carefully by manual compression and suction of the abdomen at the end of the procedure through the trocar. The patients were reversed with intravenous neostigmine and glycopyrrolate and extubated. All patients were transferred to the post-anesthesia care unit following extubation.

The intensity of postoperative pain was recorded for all patients using the visual analog scale (VAS) score at 0, 2, 4, 6, 12, and 24 hours, with 0 referring to the time the patient was transferred to recovery. In patients that complained of moderate to severe pain, 100 mg of tramadol was administered IV as a rescue analgesia treatment, and the time interval to the first request for rescue analgesia was noted. Adverse effects of nausea, vomiting, and shoulder pain over the course of 24 hours were noted. All postoperative findings were compared between the two study groups.

Data were collected, tabulated, and coded, and then analyzed using repeated-measures analysis of variance (ANOVA), the student's t-test, and the paired t-test. The Statistical Package of the Social Sciences (SPSS) version 15 for Windows (SPSS Inc., Chicago, IL) was employed for the data analysis. A difference with a p level of < 0.05 was considered statistically significant.

## Results

All the patients in Group B and Group N were comparable in terms of age, weight, sex, and the duration of surgery (Table 1).

**Table 1:** Demographic characteristic of the patients in the two study groups

	Age	Weight	Sex	Duration of Surgery
Group N (mean)	40.13	77.83	M (19) F (11)	62.16±15.18
Group B (mean)	39.8	76.73	M (19) F (11)	64.66±15.64
P-value	0.85	0.67		0.09

The VAS score was assessed at zero hours (the time when the patient was transferred to recovery) and at two, four, six, 12, and 24 hours. The mean VAS score in Group B was 1.56 at zero hours, 1.99 at two hours, 2.11 at four hours, 2.45 at six hours, 3.36 at 12 hours, and 3.53 at 24 hours. The mean VAS score in Group N was 3.46 at zero hours, 3.83 at two hours, 3.90 at four hours, 4.22 at six hours, 4.32 at 12 hours, and 4.38 at 24 hours. The VAS score was statistically significantly lower in Group B at zero, two, four, and six hours in comparison to Group N; the P-value was 0.05 (0 hours), 0.04 (2 hours), 0.04 (4 hours), and 0.04 (6 hours). The VAS score between the two groups did not show any significant difference at 12 hours (P = 0.1) and 24 hours (P = 0.7) (Table 2).

**Table 2:** Postoperative visual analog scale score and analgesia requirements

	VAS score 0 hours	VAS score 2 hours	VAS score 4 hours	VAS score 6 hours	VAS score 12 hours	VAS score 24 hours	Time taken to request rescue analgesia
Group N (mean)	3.46±1.27	3.83±1.39	3.90±1.21	4.22±1.26	4.32±1.14	4.38±1.17	70.44±24.73
Group B (mean)	1.56±0.89	1.99±0.92	2.11±0.95	2.45±1.11	3.36±1.18	3.53±1.04	182.83±24.05
P value	0.05	0.04	0.04	0.04	0.1	0.7	0.04

The mean time taken for the first requirement for rescue analgesia was 182.83 minutes in Group B and 70.44 minutes in Group N, which was statistically significant (P = 0.04) (Table 2).

In Group N, postoperative nausea occurred in two patients and vomiting occurred in three patients. In Group B, nausea occurred in one patient and vomiting occurred in two patients. The incidence of PONV was not statistically significant between the groups (P = 0.1 and p = 0.09, respectively). Shoulder pain was seen in 17 patients in Group N and seven patients in Group B, which was statistically significant (P = 0.04) (Table 3).

**Table 3:** Postoperative side effects

	Group N (n=30) (%)	Group B (n=30) (%)	P-value
Nausea	2 (6%)	1(3%)	0.1
Vomiting	3(10%)	2(6%)	0.09
Shoulder Pain	17(56%)	7(23%)	0.04

## Discussion

Laparoscopic cholecystectomy is a widely performed, common, elective procedure. Postoperative pain is comparatively less after laparoscopic cholecystectomy than after an open cholecystectomy, but it still remains a significant cause for morbidity [12]. Patient education regarding the degree of pain they may encounter, an explanation of the pain assessment tools being used and the modalities of pain treatment available, and reassurance regarding the pain they may experience can help reduce the patients’ anxiety and postoperative pain incidence.

Patients undergoing laparoscopic cholecystectomy suffer considerable pain on the day of surgery, frequently requiring opioid analgesia. After laparoscopic cholecystectomy, several types of pain can arise. Parietal pain is due to the placement of trocars through the abdominal wall. It is superficial and can be located by the patient. Visceral pain is due to intraperitoneal dissection and insufflation of CO_2_, resulting in distension of the abdominal wall. This type of pain is dull, more diffuse than parietal pain, and difficult to locate. The prolonged elevation of the diaphragm and residual gas from pneumoperitoneum leads to referred shoulder tip pain. Normal saline infiltration reduces visceral pain and referred pain by causing dilution of the inflammatory markers, replacing the gas above the liver, and reducing the space between the liver and the diaphragm [13]. Bupivacaine is the most common local anesthetic used intraperitoneally for postoperative pain relief because of its high potency and longer duration of action.

The demographic data were similar in both study groups. The overall duration of surgery was similar for both groups. In our study, the VAS score was lower in Group B (receiving intraperitoneal instillation of bupivacaine) in comparison to Group N (receiving normal saline). This finding was similar to the results reported in Devalkar and Salgaonkar [14] and Suma and Vikranth [15]. Raetzell et al. compared lower concentrations of bupivacaine (0.125% and 0.25%) with normal saline and found no difference in the pain scores between the groups [16]; this could be attributed to the lower concentration of bupivacaine that was used. In the meta-analysis by Choi et al., which studied 39 random control trial reviews, the authors concluded that intraperitoneal local anesthetics did not significantly reduce parietal pain and exhibited a favorable analgesic effect towards visceral pain and shoulder pain [17].

There are a few studies in which the administration of a local anesthetic did not show any efficacy. These failures could be due to the use of a lower drug dose, a lower concentration, or because the entire dose was infiltrated under the right hemidiaphragm [16,18-19].

In our study, postoperative shoulder tip pain was lower in Group B than Group N. Putta et al. also showed that the incidence of shoulder pain was significantly reduced in groups receiving bupivacaine in comparison to those receiving normal saline and that the timing of the bupivacaine infiltration (pre-emptive or postsurgical) was not significant [20].

In our study, the mean time taken for the first dose of analgesic was 182 minutes in Group B and 70 minutes in Group N. Similarly, the study by Sulekha showed that the dosing of rescue analgesia was more frequent and the highest in patients that received normal saline in comparison to those that received bupivacaine [21]. A study on laparoscopic pelvic surgery conducted by Shalan et al. found that the pain score and required analgesic dose were lower in the bupivacaine group [22].

No statistically significant difference was found in the incidence of PONV in Group B (one and two patients, respectively) and Group N (two and three patients, respectively). No side effects or toxicity were recorded due to bupivacaine use. Hazinedarogle et al. reported the same results [23]. Goldstein A et al. showed a lower rate of PONV in patients that were administered bupivacaine [24].

## Conclusions

In our study, we found that bupivacaine reduces postoperative pain and prolongs the requirement time of rescue analgesia. It also reduces the incidence of shoulder pain after laparoscopy. We conclude that the intraperitoneal and subdiaphragmatic instillation of 0.5% bupivacaine is a safe and effective method for providing postoperative analgesia without significant side effects.
